# The source of microbial transmission influences niche colonization and microbiome development

**DOI:** 10.1098/rspb.2023.2036

**Published:** 2024-02-07

**Authors:** Isabel S. Tanger, Julia Stefanschitz, Yannick Schwert, Olivia Roth

**Affiliations:** ^1^ GEOMAR, Helmholtz Centre for Ocean Research Kiel, Marine Evolutionary Ecology, Düsternbrookerweg 20, 24105 Kiel, Germany; ^2^ Zoological Institute, Marine Evolutionary Biology, Kiel University, Am Botanischen Garten 1-9, 24118 Kiel, Germany

**Keywords:** microbial colonization, vertical transmission, male pregnancy, early life microbiota, metaorganism, microbiome

## Abstract

Early life microbial colonizers shape and support the immature vertebrate immune system. Microbial colonization relies on the vertical route via parental provisioning and the horizontal route via environmental contribution. Vertical transmission is mostly a maternal trait making it hard to determine the source of microbial colonization in order to gain insight into the establishment of the microbial community during crucial development stages. The evolution of unique male pregnancy in pipefishes and seahorses enables the disentanglement of both horizontal and vertical transmission, but also facilitates the differentiation of maternal versus paternal provisioning ranging from egg development, to male pregnancy and early juvenile development. Using 16S rRNA amplicon sequencing and source-tracker analyses, we revealed how the distinct origins of transmission (maternal, paternal and horizontal) shaped the juvenile internal and external microbiome establishment in the broad-nosed pipefish *Syngnathus typhle*. Our data suggest that transovarial maternal microbial contribution influences the establishment of the juvenile gut microbiome whereas paternal provisioning mainly shapes the juvenile external microbiome. The identification of juvenile key microbes reveals crucial temporal shifts in microbial development and enhances our understanding of microbial transmission routes, colonization dynamics and their impact on lifestyle evolution.

## 1. Introduction

The coevolution of the host with its microbiome permitted the intimate physical integration of microbes shaping development, nutrition and digestion [1–3], but also influencing behaviour [4] and immune functions [5–7]. Microbes may provide their hosts with evolutionary novelties facilitating the co-option of new lifestyles and the colonization of new habitats [8]. Often these microbial communities are highly species, tissue and development stage-specific, others may form complex long-lasting interactions with their host [9,10]. Acquisition of specific microbes can be of horizontal source (environmental or close contact with conspecifics), vertical through gestation or incubation, or a mix of both transmission modes [8,11,12]. In horizontal transmission, interests of host and microbe may frequently oppose each other fostering antagonistic interactions [13]. This is in stark contrast to vertical transmission, where the transmission of the microbe is directly linked to the reproductive success of its host favouring cooperation [12–15] and ensuring consistent transmission of the same lineage of microbe across generations [16]. For the host and its symbiont to evolve as a unit, the coexistence of the host–microbe association with matching host–microbe genotype on evolutionary timescales is a prerequisite [13,16]. Vertical transmission not only facilitates coexistence across host generations [17,18] but the coevolution of host and microbe can evoke closer host dependence, as demonstrated over the loss of host fitness upon removal of vertically versus horizontally transmitted symbionts [16]. Even as a temporary partner with only a small transient contribution, a vertically transmitted microbe was proposed to be able to play out its evolutionary role [8].

To date, only a few studies have assessed the microbial communities of parents, gametes and offspring in depth [19–25]. Vertical transmission pathways are numerous and range from the deposition of microbes into the oocytes [26,27] or embryos [28,29], over smearing of microbes onto the egg during oocyte development and oviposition [30,31], to a variety of intimate parental–offspring interactions, e.g. pregnancy, birth and physical contact [7,32–35]. The multitude of vertical transmission routes, and the fact that most transmission routes are intermingled within the maternal line (but see: [20]) make the study of vertical transmission demanding, hindering the disentanglement of synergistic, additive or antagonistic transmission dynamics and their impact on the host physiology.

Early niche colonizers prime and boost the immature vertebrate immune system [36] and support host humoral and cellular immune defence by competing against potentially virulent microbes. These early colonizers thus prevent detrimental microorganisms from attachment, replication and colonization [11,37–39], and aid the immune system in learning to differentiate friend from foe in order to maintain symbiotic relationships [40,41].

To understand the development of the microbial community, we need to unravel the source of the microbial symbionts and assess niche colonization and expansion during different developmental stages. Spotting early colonizers inherited from parents through vertical transmission routes is particularly crucial. Mothers transfer microbes to their offspring during egg development, pregnancy or postnatal brood care. In lifestyles with intense father–embryo contact, alternative routes for vertical microbial transmission can be identified. Teleost fishes show the most extensive variation in parental care [42]; 20% of bony fishes exhibit an intimate parent–embryo contact during embryo development [43], of these half display exclusive male care [44–46]. The unique male pregnancy evolution in pipefishes, seahorses and seadragons (the syngnathids) offers differentiating the often-intermingled routes of vertical transmission. In the broad-nosed pipefish *Syngnathus typhle* [47,48] the embryos are connected to the paternal body and are supplied with nutrients, oxygen and immunity via a placenta-like organ [49–52]. The larval mouth opening develops only in the second half of pregnancy (17 days post-mating) [53]. Pipefishes are exposed to a diversity of microbes and harbour a sex-specific microbiome [19,51]. The difference in the microbiome of the female ovaries and the male brood pouch offers the opportunity for vertical transfer of the maternal microbiome throughout the oocytes, while the paternal microbiome may be transferred to the embryo during male pregnancy [19]. Understanding the contribution of maternal (via egg), paternal (during pregnancy) and horizontal (environmental) microbes to microbial colonization of offspring will significantly advance our understanding of how the usually maternally intermingled routes of vertical transmission, i.e. egg production and pregnancy, play out and interact in the establishment of the juvenile microbiome.

To unravel how the maternal versus the paternal routes of vertical transmission, as well as the horizontal route drive embryonal and juvenile microbiome development, we conducted two experiments with the broad-nosed pipefish spanning egg development throughout male pregnancy and ending with the first days post-juvenile release. The first experiment aimed to identify differences in microbial community composition, as well as define key microbes and track microbial source before fertilization and during male pregnancy. To determine how the route of microbial transmission (maternal, paternal and horizontal) shapes microbiome establishment in distinct offspring niches, we genotyped microbial 16S rRNA in unfertilized eggs (surface sterilized or unmanipulated), male and female hindguts, male brood pouches, testes and juveniles (surface sterilized and unmanipulated) at three time points during pregnancy. The comparison of internal (surface sterilized) and external microbial community in unfertilized eggs and developing embryos permitted to elucidate the origin of organ-specific microbial communities during embryogenesis and pregnancy and their contribution to the establishment of internal versus external microbiome. In the second experiment, we assessed the microbial community of juvenile broad-nosed pipefish during the first 12 days post-release (dpr) from the brood pouch. Inferring about gut versus whole-body microbiome, this experiment illuminated the development of the microbiome from birth throughout the first environmental microbial contact, highlighting how the supposedly maternally and paternally vertically transmitted microbes play out when horizontal transmission routes take over in establishing the pipefish microbiome. This study permits pinpointing specific microbes in the parental organs, over male pregnancy, and assess their contribution to offspring microbial colonization. The insights will advance our knowledge about how routes of vertical transmission interact in microbial colonization and establishment, and ultimately unravel how microbes influence lifestyle evolution.

## 2. Methods

### (a) Experimental design and sample collection

Adult *Syngnathus typhle* were caught in Orth on Fehmarn (54°26′N 11°02′E) and brought to our aquaria facilities at GEOMAR Kiel, for breeding. Fish were kept in a flow-through aquaria system at 18°C with 18 h day/6 h night light regime and fed with live and frozen *Mysidae* spp. twice a day. In each aquarium three males and three females were kept together to allow mating. After the onset of breeding, fish were randomly sampled, regarding their sex and gravity stages, on 5 days between the end of May 2019 and end of June 2019. We sampled 89 mature *S. typhle* (18 females, 16 early pregnant males, 19 mid-pregnant males, 18 late pregnant males and 19 non-pregnant males), at each time point one male was taken from each aquarium. Pregnancy stages (early, mid and late pregnancy) were defined according to [53]. To detect microbial transfer from parental gonads and pouch tissue to the juveniles, we sampled testes and endometrial inner pouch lining tissue as well as fertilized larvae from the three pregnancy stages in male fish. In female fish, we sampled unfertilized eggs to assess potentially deposited microbes into the eggs or on their surface. The hindgut was sampled irrespective of sex. To investigate maternal microbial transfer through the cytoplasm, we surface sterilized half of the unfertilized eggs from each female in 0.5% polyvinylpyrrolidone-iodine (PVP-I, solution in sterile-filtered phosphate buffered saline (PBS)) for 5 min with subsequent washing three times with 500 µl sterile-filtered PBS (adapted from [54]). To sample the cytoplasm of surface sterilized eggs (sterilized eggs), the egg was squashed in the collection tube. The same sterilization treatment was applied to larvae (sterilized juveniles) of different pregnancy stages to discriminate between the external and the internal microbiome. Non-sterilized eggs (untreated eggs) and larvae (untreated juveniles) were directly placed in the collection tubes. We pooled three juvenile and egg samples from each of the pregnancy stages and sterilization treatment. All sampled organs were collected in microtubes from the DNeasy96 Blood and Tissue Kit from Qiagen (Hilden, Germany) and stored immediately at −80°C (details: electronic supplementary material S1).

### (b) Juvenile microbiome development

Pregnant male *S. typhle* were caught in Orth on Fehmarn (54°26′N 11°02′E), transferred to the aquaria system, kept individually in a flow-through system with 18 h day/6 h night cycle and fed twice a day with live and frozen *Mysidae* spp. After parturition, free-swimming juveniles of each male were kept in a distinct aquarium and fed *ad libitum* twice per day with live *Artemia salina*. First sampling took place after release from the brood pouch before first feeding, sampling was continued in 2–3 day intervals. For each sampling three juveniles per family tank were collected individually in a collection tube for the analysis of whole-body microbiome (whole juveniles) development. To test for development of gut versus whole-body microbiome another three juveniles from the same family tank were killed by brain section and the gut was removed in a sterile manner (juvenile gut) and collected individually. At each sampling day, controls of water and food samples were taken.

### (c) RNA extraction, library preparation and amplicon sequencing

Both datasets have been treated with the same DNA extraction and 16S rRNA sequencing protocols (DNeasy Blood & Tissue Kit (QIAGEN, Germany)) following the manufacturer's protocol including a pre-treatment for Gram-positive bacteria with ameliorations from [55] (electronic supplementary material S2). Library preparation was done by the Institute for Experimental Medicine (UKSH, Campus Kiel) with 20 µl DNA from each sample. Amplicons of the V3-V4 hypervariable region (341f/806r) were sequenced using the Illumina MiSeq platform (Illumina, USA) with 2x 300 bp paired-end read settings at the Institut for klinische Molekularbiologie (IKMB), Kiel University.

### (d) Data analysis

Demultiplexed sequences were processed using DADA2 implemented in the Qiime2 platform (version 2021.8 [56]) for primer cutting, timing, quality control, merging, chimera removal and denoising. Taxonomy was assigned using the Silva 132 classifier for Qiime 2 (version 2019.10) for the V3/V4 hypervariable region. Mitochondrial and chloroplast sequences were removed before further analyses. Sorting and statistical analysis of exported Amplicon Sequence Variants (ASV) were conducted in R (version 4.1.0, [57]) using the phyloseq package (version 1.36.0, [58]). The two experiments were analysed separately using the same parameters. After removal of ASVs with non-definite taxonomic classification (NA) in phylum, family and genera, we applied a prevalence filter of 2% and agglomerated the sequences on genus level, a separate analysis on ASV level has been conducted.

Faith PD, Shannon index and observed number of genera represent α-diversity (‘microbiome’, version 1.14.0, [59]). Hypothesis testing using an aov (‘stats’, version 4.1.0, [57]) with either blocked ANOVA for the adult treatment (x∼gravity + organ, data = adult) or a repeated measures ANOVA for the juvenile dataset (x ∼ treatmentAW * timepointAW, strata = familyAW, data = juvenile). In the case of significant ANOVA effects, a Tukey honest significant difference (HSD) test using false-discovery rate was applied as a *post-hoc* test.

β-diversity was tested on a Bray–Curtis dissimilarity matrix (BCdM) and unweighted UniFrac distance matrix (UFdM). The first calculates relative abundance and infers information about the numerical composition within/between microbial communities, the latter includes phylogenetic distance between the genera providing insight into the phylogenetic spread of a microbial community. β-diversity was tested in a two factorial blocked permutational multivariate analysis of variance (PERMANOVA) (‘vegan’ version 2.5.7, [60]) (vertical transmission; adonis (x_brayCurtis∼organ + gravity, data = adult, permutations 10 000)) or a two factorial repeated measures PERMANOVA (juvenile development; adonis (x_brayCurtis ∼ treatmentAW * timepointA, data = juvenile, strata = familyAW, permutations 10 000)) on BCdM and UFdM. Pairwise.adonis2 (version 0.4, [61]) was used as a *post-hoc* test to detect pairwise differences. False discovery rate was used for *p*-value adjustment to multiple testing. Data were visualized using a principal coordinate analysis of the 50 most abundant genera including 95% confidence ellipses.

An indicator species analysis (ISA, (‘indicspecies’, version 1.7.9, [62])) was run on both datasets independently to identify microbial genera indicative of either single levels of each factor or level combinations. To estimate the proportion of ASVs originating from parental organs, environment or contamination controls, we applied Bayesian community-level microbial source tracking (BMST) (sourcetracker2 1.0.1, [63]).

## 3. Results

### (a) Parental transfer of microbes

To analyse vertical transfer of microbiota during pregnancy, we calculated α-diversity and β-diversity on gravity stages (female, non-pregnant, early, mid and late pregnant) and organs (paternal: testes, hindgut, placenta-like tissue and maternal: surface sterilized eggs and untreated eggs, juveniles: untreated and surface sterilized juveniles). The phylogenetic diversity and the general biodiversity were assessed using a comparison of α-diversity indices (Faith PD, Shannon and observed genera). To infer differences among the organs or gravity stages, we calculated the phylogenetically weighed UFdM. To assess microbial distribution BCdM was calculated. UFdM and BCdM permit assessment of the similarities and differences of the microbiomes among organs and gravity stages.

The BMST infers the proportion and probability of a source microbiome to be present in a sink microbiome, providing a tool to find traces of the source microbiome in the sink microbiome [64–66]. We aimed to investigate the proportion of microbes, identified in the juvenile microbiome (surface sterilized juveniles and non-surface sterilized juveniles; sink), shared with the microbiome isolated from the parental organs (paternal: placenta and testes versus maternal: surface sterilized unfertilized eggs and non-surface sterilized unfertilized eggs), with the environmental microbiome (water) and assess potential contamination through the sampling process (control: PBS / PVP-I) (all source populations). By mapping the ASVs of the sink microbiomes on the source microbiomes, we could infer the proportion of source microbiome shared with the sink microbiome. As the sink microbiome is not solely a combination of the source microbiome, source tracker analysis introduces an additional source: unknown, accounting for parts of the sink microbiomes not identified in the source microbiomes. The BMST displays how much of the juvenile microbiome originates from either parental organs, water or controls and thus hints towards vertical transfer.

During denoising and filtering 12 samples were removed from the downstream analysis. After removing sequences with no taxonomic information (NA), 2% prevalence filtering and taxonomic agglomeration, 278 unique genera have been identified. The total read number was 6′456′920 with a mean read number of 17′546 reads/ sample. In contrast, PBS samples had a mean read numbers of 3′613. The most prevalent phyla were Proteobacteria (56.83%), Bacteroidetes (19.78%), Firmicutes (8.2%) and Actinobacteria (4.68%).

α-diversity: differences in phylogenetic diversity measured by the Faith PD existed between the organs irrespective of gravity (blocked ANOVA: Faith PD genus level: organ: *F*_9,355_ = 6.741, *p* < 0.001 ***, gravity: *F*_4,355_ = 0.611, *p* = 0.655; electronic supplementary material, figure S1, table S1, relative abundance is depicted in electronic supplementary material, figure S3) (blocked ANOVA: Faith PD ASV level: organ: *F*_9,355_ = 5.803, *p* < 0.001 ***, gravity: *F*_4,355_ = 1.232, *p* = 0.297; electronic supplementary material, table S2). Additional α-diversity indices, Shannon diversity index and observed number of genera/ ASV, do not deviate from the results of Faith PD and are provided in the electronic supplementary material, figure S2 (A and B) and table S1. Sterilized eggs and sterilized juveniles had a lower phylogenetic diversity than untreated juveniles and the placenta, but no differences were found between the untreated juveniles and the placenta or testes. Further, the placenta had a higher phylogenetic diversity than untreated eggs and the testes.

**Figure 1.**
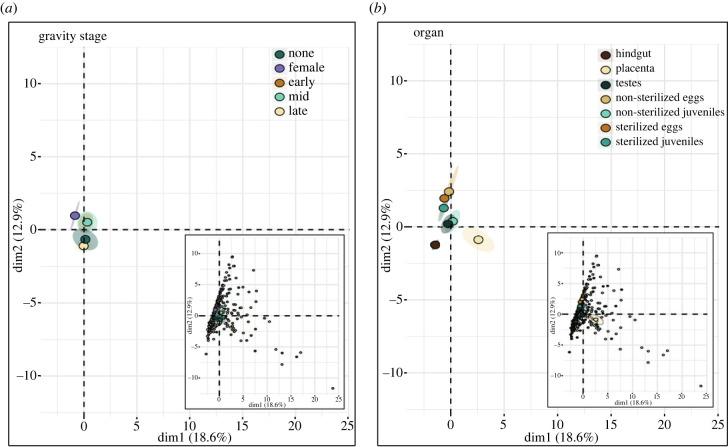
Principal coordinate analysis (PCoA) of the 50 most abundant microbial genera during *S. typhle* pregnancy. The first two principal components, Dim1 explaining 18.6% of the total variance and Dim2 explaining 12.9% of the total variance are shown together with 95% confidence ellipses (shaded area) around a centre of gravity (big points). Samples are indicated as small points within the smaller graphs (larger versions in electronic supplementary material, figure S4). (*a*) Colours represent gravity stages associated with the top 50 microbial genera. (*b*) Colours represent organ association of the top 50 microbial genera.

β-diversity: β-diversity, calculated from both UFdM and BCdM, showed effects in organs (untreated juveniles, sterilized juveniles, untreated eggs, sterilized eggs, testes, placenta and hindgut) and gravity stages (female, non-pregnant, early, mid and late pregnant) (blocked PERMANOVA; Bray–Curtis dissimilarity matrix genus level: organ: *F*_9,354_ = 6.13, *p* < 0.001 ***, R^2^ = 0.13, gravity: *F*_4,354_ = 2.31 *p* < 0.001 ***, R^2^ = 0.02; unweighted Unifrac: organ *F*_9,354_ = 4.65, *p* < 0.001 ***, R^2^ = 0.1, gravity: *F*_4,354_ = 1.57 *p* = 0.0128 *, R^2^ = 0.01) (figure 1). In the factor organ, pairwise comparisons of UFdM revealed distinct microbial composition between untreated juveniles and sterilized juveniles (*p* = 0.003), untreated eggs (*p* = 0.003), sterilized eggs (*p* = 0.003) and water (*p* = 0.041). However, a similar phylogenetic microbiome was suggested in untreated juveniles and the placenta (*p* = 0.0725). Apart from this, all organs differed in their microbial composition. Pairwise comparisons of the BCdM, showed a difference between untreated juveniles and the placenta (placenta: untreated juveniles *p* = 0.03).

Effects of gravity stage on phylogenetically weighted β-diversity (UFdM) were found between females and late, mid and non-pregnant males (female:late *p* = 0.01, f:mid *p* = 0.05, f:male *p* = 0.01). Additionally, late pregnant males differed from early pregnant males (*p* = 0.05). The non-phylogenetically weighted BCdM revealed differences between females and non-pregnant males (*p* = 0.01) and late pregnant males (*p* = 0.01), as well as differences between late pregnant males and both early (*p* = 0.01) and mid-pregnant males (*p* = 0.01). For all statistical information see electronic supplementary material, table S3 (genus level) and table S4 (ASV level).

Indicator species analysis: differences in the microbiome composition between organs or gravity stages were established with an indicator species analysis (ISA) on all 278 genera (electronic supplementary material, tables S5 and S6, electronic supplementary material S3). Genera and association to gravity stages and organs are displayed in electronic supplementary material, table S14.

**Figure 2.**
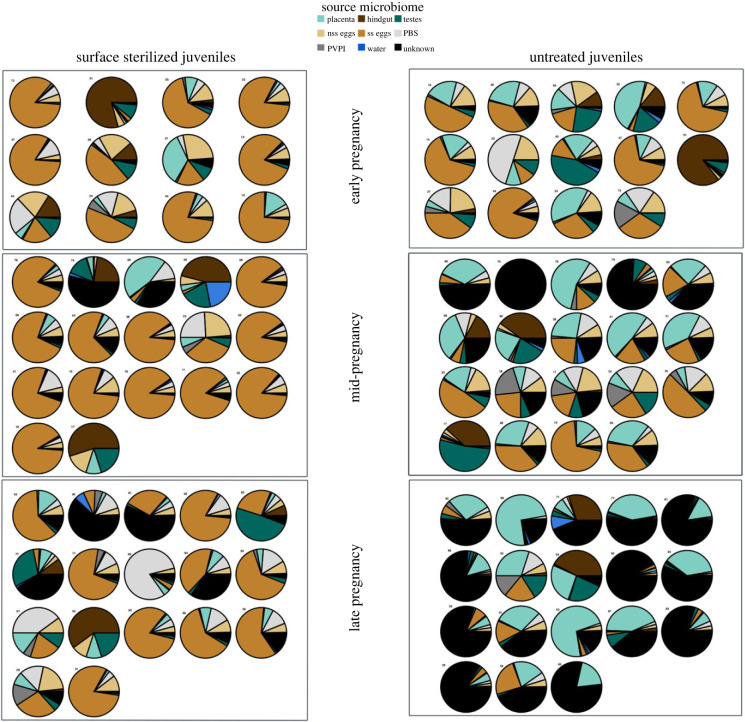
ASV-level SourceTracker analysis of the juvenile microbial community during pregnancy. Proportion of each source microbial community after 100 Gibbs samplings represented as partial circles in each sink sample. Sink samples were defined as untreated and surface sterilized juveniles during the different pregnancy stages (early, mid and late pregnancy). nss eggs = non-surface sterilized, untreated eggs/gonads and ss eggs = surface sterilized eggs/gonads.

Bayesian microbial source tracking: we conducted a BMST on ASV level computing the proportion of juvenile microbiome (sterilized juveniles and untreated juveniles) originating from a specific source microbiome (all parental organs (sterilized eggs, untreated eggs, hindgut, placenta, testes)), the environment (water) and the controls (PVP-I, PBS) (figure 2). Proportions are displayed in electronic supplementary material, table S7. BMST suggests that sterilized juvenile microbes are mostly shared with the microbiome of the sterilized eggs (maternal origin), while a smaller proportion was shared with the microbiome of the parental hindgut (male and female), the placental tissue (paternal) and of untreated eggs (maternal). The source microbiome contribution to the sterilized juveniles was stable throughout male pregnancy, whereas in the untreated juveniles, the supposed source of the microbiome changed substantially over the course of male pregnancy. In early pregnancy, the microbiome was predominantly of maternal source, as the microbiome of the sterilized eggs (unfertilized and before transfer to the paternal pouch) contributed most to the early pregnancy untreated juveniles, however, this was in tandem with an increasing microbial contribution from the testes. Throughout pregnancy, the microbiome of the untreated juveniles changed from known sources of either sterilized or untreated eggs, placenta, testes and hindgut to an unknown source of microbiome. This unknown source represents a proportion of the untreated juvenile microbiome which is not found in either of the designated source microbiomes (maternal and paternal organs, controls, environment). In late pregnancy this unknown source contributed highest to the untreated juvenile's microbiome, followed by placenta, testes and untreated gonads. The proportion of microbes with a suggested source in seawater and the controls for PVP-I and PBS remained low in all samples.

We could identify a clear distinction in the microbiome of untreated juveniles and surface sterilized eggs, untreated eggs and surface sterilized juveniles, whereas untreated juveniles and the placenta share a similar microbiome as suggested by the α- and β-diversity. The BMST indicated more maternally originated sources in surface sterilized juveniles and more paternally originated sources in untreated juveniles. Additionally, the proportion of unaccountable sources (unknown) rise during the course of pregnancy in untreated juveniles.

### (b) Juvenile microbial community development

We applied α- and β-diversity measures to determine the establishment of the microbial community in juveniles depending on timepoint (dpr) and treatment (16S rRNA from whole body or gut in intervals of 2–3 days).

After removing sequences with no taxonomic information (NA), 2% prevalence filtering and taxonomic agglomeration at genera level, we identified 495 unique genera. The total read number was 5′462′385 with a mean read number of 14′566.36 and a median of 13′644 reads. The most prevalent phyla over all samples were Proteobacteria (48.29%), Bacteroidetes (16.97%), Actinobacteria (6.27%) and Firmicutes (6.06%).

α-diversity: we used Faith PD as an α-diversity measure between whole-body and gut microbiota over sampling time. The repeated measures ANOVA (strata = family) showed an interactive effect of time and treatment (whole body or gut) on α-diversity as well as a significant treatment effect (repeated measures ANOVA Faith PD genus level: treatment: *F*_1,351_ = 1314.708, *p* < 0.001 ***, timepoint: *F*_6,351_ = 1.973, *p* = 0.068 interaction terms: *F*_6,351_ = 3.341, *p* = 0.0033 **). *α* -diversity indices are provided in the electronic supplementary material, figures S5 and S6 (A and B) and table S8 (genus level) and 9 (ASV level), relative abundance in electronic supplementary material figure S7. Faith PD showed a higher phylogenetic diversity in whole-body juvenile microbiome compared with juvenile gut (*p* < 0.001), as well as differences in microbiomes of water and juvenile samples (water-whole body *p* = 0.0019, water-juvenile gut *p* < 0.001) and differences between artemia and whole-body juveniles (*p* < 0.001). No significant differences in Faith PD of the gut microbiome and artemia were detected (*p* = 0.224). The phylogenetic microbiome diversity was not affected by sampling timepoint.

**Figure 3.**
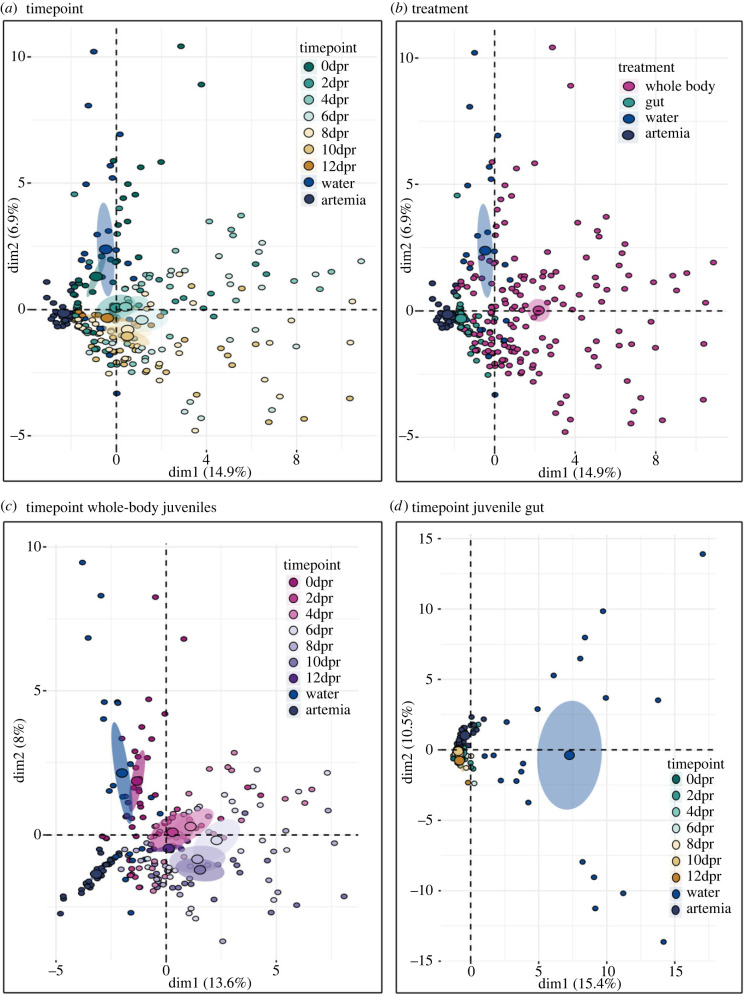
Specific principal coordinate analysis (PCoA) of microbial genera during juvenile development. First two principal components of the top 50 most abundant genera with 95% confidence ellipses (shaded area) around a centre of gravity (big points). Samples are indicated as small points. (*a*) and (*b*) Dim1 explains 14.9% of the total variance and Dim2 explains 6.9%. (*a*) PCA assessing the effect of temporal change on the top 50 microbial genera during *S. typhle* juvenile development irrespective of treatments. (*b*) PCA displaying the effect of sampled organ, juvenile whole body or juvenile gut, on the top 50 microbial genera during *S. typhle* juvenile development irrespective of sampling timepoints. (*c*) PCA displaying the temporal change on the top 50 microbial genera in juvenile whole-body microbiome. Dim1 explains 13.6% of the total variance and Dim2 explains 8%. (*d*) PCA displaying the temporal change on the top 50 microbial genera in the juvenile gut microbiome. Dim1 explains 15.4% of the total variance and Dim2 explains 10%.

β-diversity: we computed a two factorial repeated measures PERMANOVA on BCdM and UFdM using timepoint of sampling and treatment as factors with family as strata. We identified differences in β-diversity between microbial communities in juvenile gut and whole-body juvenile over time in accordance with the influence of family on β-diversity (repeated measures PERMANOVA: BCdM genus level: treatment: *F*_3,359_ = 34.08, R^2^ = 0.2007, *p* < 0.001 ***, timepoint: *F*_6,351_ = 5.146, R^2^ = 0.061, *p* < 0.001 ***, interaction terms: *F*_6,351_ = 2,875, R^2^ = 0.034, *p* < 0.001 ***; UFdM: : treatment: *F*_3,359_ = 73.198, R^2^ = 0.353, *p* < 0.001 ***, timepoint: *F*_6,351_ = 4.276, R^2^ = 0.0413, *p* < 0.001 ***, interaction terms: *F*_6,351_ = 2,785, R^2^ = 0.027, *p* < 0.001 ***) figure 3. According to *post-hoc* testing in BCdM, differences between whole-body juveniles and juvenile gut irrespective of the timepoint sampled were identified (whole body (0dpr–12dpr): gut (0dpr–12dpr) *p* < 0.001). The microbial β-diversity of whole-body juveniles differed among all timepoints (*p* < 0.01, see electronic supplementary material, table S10 (genus level)) except from 2dpr to 4dpr (*p* = 0.1169), 4dpr–6dpr (*p* = 0.0627), 6dpr to 8dpr (*p* = 0.0578) and 8dpr–10dpr (*p* = 0.2269). The β-diversity of the juvenile gut microbiome differed between day of release and all other sampling timepoints (gut 0dpr: gut (2–12dpr) *p* < 0.01), further differences were found between 2dpr and 4dpr (*p* = 0.0336), 2dpr and 6dpr (*p* = 0.0137) and 2dpr and 10dpr (*p* = 0.0036). Samples from 4dpr differed in their β-diversity from samples from 10dpr (*p* = 0.0048) and samples from 4dpr and 6dpr differed from 12dpr (4dpr: 12dpr: *p* = 0.0137, 6dpr: 12dpr: *p* = 0.0249). Additionally, β-diversity of the juvenile gut microbiome between 8dpr to 12dpr differed (*p* = 0.0389). All samples, irrespective of timepoint and treatment had a distinct β-diversity than both water (*p* < 0.01) and artemia (*p* < 0.01) (electronic supplementary material, figure S10). Similar results were found in the *post-hoc* test of the phylogenetically weighted UFdM analysis. In contrast to the BCdM results, there were significant differences in the whole-body juveniles between 2dpr and 4dpr (*p* = 0.029) and between 4dpr and 6dpr (*p* = 0.0095). However, the juvenile gut microbiome was more constant over time. As such, 2dpr did not differ from any other timepoint except 10dpr (*p* < 0.05) and the microbial β-diversity of the 8dpr juvenile gut was similar to the microbial β-diversity of the 12dpr juvenile gut. ASV-level analyses are provided in electronic supplementary material, table S11.

Indicator species analysis: to assess key microbial genera within treatments or timepoints, we conducted an ISA over all 495 genera (electronic supplementary material, tables S12 and S13, electronic supplementary material S4). Microbial communities differed between whole-body and gut samples already at the day of release. Further, the whole-body microbiome changed more over time than the gut microbiome. Crucial timepoints for shifts in β-diversity were 0dpr–2dpr and between 2 and 4dpr–6,8,10dpr.

### (c) Early life key microbial genera

In order to find key microbial genera for vertical transfer and juvenile microbiome development, we conducted indicator species analyses. For each factor in both datasets, we conducted individual ISAs in order to identify key genera represented in adult organ, adult gravity, juvenile treatment and juvenile timepoint. Genera present in two or more of the four ISAs are considered important during pregnancy and early microbiome development in juveniles (electronic supplementary material, table S14). Three bacterial genera (*Corynebacterium, Streptococcus, Yersinia*) were prevalent both in the gut of free-swimming juveniles and sterilized eggs (electronic supplementary material, table S14). *Planktotalea, Dokdonia, Croceitalea* and *Candidatus Megaira* were found in untreated juveniles, placenta and whole-body microbiome. Nine genera (*Lewinella, Sulfitobacter, Thalassotalea, Neptumononas, Crocinitomix, Owenweeksia, Ulvibacter, Kiloniella* and *Fabibacter*) were present in male reproductive organs, untreated juveniles and whole-body juveniles. These have earlier been identified as vertical transmission candidates (Beemelmanns *et al*. [19]). *Croceitalea* and *Kiloniella* were found in late pregnancy and during the first days post-release. *Sphingorhabdus*, *Defluviimonas* and *Flavobacterium* were identified in placenta and whole-body microbiome of juveniles, but not in untreated juveniles (electronic supplementary material, table S1).

## 4. Discussion

To unravel how the maternal versus paternal routes of vertical transmission and the environmental contribution via the horizontal route drive embryonal and juvenile microbiome development in the broad-nosed pipefish *Syngnathus typhle*, we have assessed 16S rRNA microbial diversity in a range of maternal and paternal organs, and in different stages of the developing offspring, as well as in the environment on both surface sterilized and untreated eggs/ juveniles. Sterilization treatment permitted to discriminate internally from externally transferred microbes.

In the first part, we evaluated changes in microbial composition throughout reproduction and male pregnancy comparing the developing larval microbiome to the microbiome of an unfertilized maternal egg, the paternal testes and the paternal placenta-like tissue of *S. typhle* [1]. To determine the maternal microbial contribution, we compared the whole-egg microbiome (untreated eggs), the egg-internal microbiome (surface sterilized eggs) and both surface sterilized and untreated juveniles at three timepoints during pregnancy. To detect paternal microbial origin, we compared the microbiome of the placenta-like structure and testes of non-pregnant males to surface sterilized and untreated juveniles at three timepoints throughout pregnancy. Further, surrounding waters were assessed to determine the environmental (horizontal) microbial contribution to the offspring. In the second part, we sampled juveniles (full sibs) post-release to understand microbial establishment [2]. We differentiated between the juvenile gut microbiome and the whole-body juvenile microbiome to resolve the role of environmental and parental microbiome in the development of the microbiome. In the third part, we identified key microbial genera both during pregnancy and early microbiota development in free swimming juveniles that should be studied in future microbial manipulation experiments [3].

### (a) Parental transfer of microbes

To assess offspring microbiome development across the gravity stages, microbial compositions in females, non-pregnant, early, mid and late pregnant males are discussed first disregarding organ specificity. While microbial α-diversity was not affected by gravity stage, β-diversity differed between gravity stages both in phylogenetic (UFdM) and compositional (BCdM) microbial diversity. Late pregnant microbial composition differed from the early and mid-pregnancy indicating a shift in microbial composition towards the end of pregnancy. This shift supports previous insights into the parental microbiome of *S. typhle* [19] and matches a similar pattern in the human vaginal microbiome throughout the mammalian pregnancy [67]. The transfer of eggs into the brood pouch influenced its microbial composition as suggested by an undistinguishable UFdM between female eggs and early pregnant male in contrast to significant phylogenetic microbial differences among female eggs and mid, non and late pregnant males.

To gain insight into organ-specific microbes, we compared the microbial community composition across organs (placenta-like tissue, testes, hindgut, sterilized and unsterilized eggs, sterilized and unsterilized juveniles) irrespective of the stage of pregnancy. The placenta-like tissue had a higher α-diversity than any other organ except untreated juveniles (electronic supplementary material, figure S1). This high microbial diversity in the placenta-like structure as the central brooding organ, might be adaptive by protecting the highly vulnerable developing juveniles from potentially virulent microbes [68]. This is essential at the onset of pregnancy when eggs are transferred into the brood pouch, and during the last third of the pregnancy when the brood pouch becomes permeable and thus sensitive to environmental influences [27]. As the place of paternal vertical transfer, a high microbial diversity in the placenta-like tissue might be favourable for the unborn juveniles, permitting a diverse initial microbial colonization. Evidence for such vertical transfer can be found in the similar Faith PD Index and phylogeny incorporating UFdM of both the placenta-like system and untreated juveniles hinting towards a phylogenetically similar microbiome (electronic supplementary material, figure S1 and figure 1*b*). No such grouping was identified in the non-phylogenetically BCdM indicating similar genera with distinct numerical compositions in the placenta-like tissue and untreated juveniles. The paternally influenced juvenile microbial composition still is subject to development given numerical proportions of microbial genera, possibly influenced by the presence of maternally transferred genera.

Maternal microbial transfer was assessed comparing untreated juveniles with both surface sterilized juveniles and sterilized/ unsterilized eggs. Considering that mouth opening only develops in the second half of pregnancy [69], previous paternal vertical transfer to the internal microbiome of the juvenile is unlikely. Microbial communities identified in the surface sterilized juvenile might thus likely have maternal origin. The possibility of maternal transovarial transfer of the microbiome was supported by a similar microbiome in both untreated and sterilized eggs and sterilized juveniles as suggested in both *α-* and β-diversity (electronic supplementary material, figure S1 and figure 1*b*). In sterilized juveniles, the BMST identified a microbial contribution from sources that consisted across the pregnancy stages (sterilized and untreated eggs) (figure 2). This permits speculation that transovarial maternal microbes contribute to the initial gut microbiome colonization before first feeding. Opposed to the rather constant microbial composition of the surface sterilized juveniles, the microbiome of the untreated juveniles, underwent severe changes throughout pregnancy regarding the source of the microbiome. The similar microbial contribution patterns of surface sterilized and untreated juveniles in early pregnancy suggest a delay in paternal vertical transfer through the placenta-like system at the onset of pregnancy. Over the course of pregnancy, a shift in the microbiome source of the untreated juvenile is indicated by a rising proportion of paternally originated microbiome (placenta-like tissue) and decreasing influence of maternal source (eggs). Following the hatching of the pipefish embryos in the brood pouch [53], the paternal microbial influence on the juveniles must have increased (figure 2), supporting our previous results [19]. In late pregnancy, to accommodate for juvenile growth, the brood pouch becomes permeable, simultaneously, the mouth opening develops, two factors that increase the probability of horizontal microbial transmission to the developing juveniles from, e.g. environmental seawater [19]. While the microbiome of untreated juveniles could be sourced by paternal organs in early and mid-pregnancy, untreated juveniles in late pregnancy exhibit a higher proportion of unknown microbial source. This indicates a contribution of a microbial community not accounted for by the source microbiomes (paternal, maternal, environmental and control) assessed in this study. A possible explanation for this rising proportion of unknown source microbiome is the development of a juvenile-specific microbiome differing in its proportion and composition from the parental and the environmental microbial community (figure 2). Our data show that the internal microbiome (surface sterilized juveniles) is sourced by the internal egg microbiome (surface sterilized eggs), the overall microbiome (untreated juveniles), however, is more diversely sourced and tends towards a stronger paternal contribution (placenta) or possibly a more unknown microbiome. This suggests that transovarial microbial transfer incorporated an important role in the establishment of the internal juvenile microbiome, potentially developing further into the gut microbiome. In contrast, paternally transferred microbial communities rather contributed to the external juvenile microbiome, potentially having a priming and protective effect on the unborn juvenile, with a gradually decreasing influence towards parturition.

### (b) Juvenile microbial community development

During pregnancy, the brood pouch microbiome, in tight interplay with the paternal immune system, is protecting the highly vulnerable juveniles from exposure to virulent infections. At birth, the release of the juveniles into the surrounding waters changes the requirements on the function of the juvenile microbiome [19]. These environmental microbes are then the main source of colonization and are supposed to shape the offspring microbiome and immune system. The second part of our study provides insights into the gut versus the whole-body microbiome of pipefish juveniles over the first 12 days post-release from the brood pouch.

Even in freshly released juveniles, the internal gut microbiome differed from the whole-body microbiome (figure 3*b*, see also electronic supplementary material, table S12 for statistical evidence). This suggests that vertical transmission contributes to initial colonization of juveniles, and emphasizes that vertical transmission is crucial for early microbial niche colonization and the establishment of an initial gut microbiome in *S. typhle* juveniles. The β-diversity of the gut microbiome of newly released juveniles was lower and shows less change over early juvenile development compared with the whole-body microbiome (electronic supplementary material, figure S8 and figure 3*b*). Most of the differences in the interaction term could be assigned to the changes of the whole-body microbial community during early free-living development (figure 3*c,d*), indicating a higher influence of horizontally transferred microbiota on the whole-body microbiome during the first few days after paternal release. An important source for microbial horizontal transfer might be provided by the artemia the juveniles were feeding on.

In the whole-body juvenile microbiome, we detected two critical microbial shifts after parturition (figure 3*c*). The first shift in β-diversity occurred between 0 and 2 dpr, supposedly imposed by the first contact to seawater and the start of feeding. At this stage, the microbiome of the whole-body juvenile was further enriched by horizontally transmitted microbes of food and seawater. The second shift occurred between 2 and 4dpr and 6, 8 and 10dpr. Cyanobacteria and Acidobacteriota were key bacteria groups defining the microbial community of freshly released juveniles (electronic supplementary material, table S13). In contrast, the juvenile microbiome 6–10dpr was defined by the genus *Rubitalea* from the phylum Verrucomicrobiota, interestingly, a genus also abundant in the hindgut of adult *S. typhle* and in untreated juveniles during pregnancy. An acclimatization phase of the internal microbiome to environmental microbes (food and water) could explain why *Rubitalea* is present in the juvenile during pregnancy but not in the first dpr. The decrease in intraindividual variation of the whole-body microbiome at 12dpr is explained by the establishment of a stable core microbiome as represented by a set of microbial taxa shared by most *S. typhle,* and supported by cases in the human microbiome [70]. A core microbiome can be temporally induced and remain stable over a certain life stage such as, e.g. pregnancy or juvenile development [71]. Insights into temporal core microbiomes will facilitate further investigations about the function of certain microbes [72] that are vertically transmitted [73].

### (c) Early life key microbial genera

We aimed to identify key microbial genera in *S. typhle* development that are important in the establishment of a microbiome. These will be candidate genera for future manipulation experiments permitting the identification of their functions in male pregnancy, juvenile development and immune system maturation. By comparing four different ISAs, we identified 33 bacterial genera suitable for further investigation (electronic supplementary material, table S13), 17 of which were previously described in male pipefish pregnancy as candidates for vertical transmission [19].

The intimate contact of pipefish over the placenta-like system to their fathers during male pregnancy selected for biparental trans-generational plasticity [61–64]. In this study, we have highlighted how the mother, father and the environment shape the microbiome of juveniles during pregnancy. Our results support possible vertical transfer from both parents with changing intensities over the course of the pregnancy. We provide evidence that transovarial transferred maternal microbes are a main source of the internal juvenile microbiome, which eventually establishes the gut microbiome in early released juveniles. Placental transfer from the pregnant male might have an enhanced role in shaping the external microbiome. We could identify key genera for early juvenile microbiome development, suitable for future manipulation studies.

Future studies could restrict sampling to the identified crucial timepoints, and instead, follow the juvenile microbiome development post-release for a longer period. Establishing a sterile gut dissection during pregnancy would enable an earlier differentiation between the gut and the whole-body microbiome providing more detailed insights into gut microbiota establishment and the specific impact of maternal vertical microbiota transfer. Altogether, this will provide a higher resolution of the maternal versus paternal role in microbial transfer and on the development of the juvenile microbiome. To disentangle parental (maternal versus paternal) from horizontal microbial transmission and follow niche colonization, we require data from several pipefish generations in order to find persistent members of the core microbiome. Experimental microbial manipulation and tracing key microbes from the parents to the offspring through genetic fluorescent markers will ensure their route of vertical versus horizontal transfer. Removing and adding bacterial strains and investigating their physiological impact on the host bridges the route of transfer to other physiological traits of the pipefish life, such as development and immune system.

## Data Availability

Metadata and FastQ Files relevant for this study have been deposited in a Dryad depository: https://datadryad.org/stash/share/dRwh5Yixb-JNWWOtE6Wcey0BUSp9JrUOtggIebCo5T0. Supplementary material is available online [74].
